# FusionU10: enhancing pedestrian detection in low-light complex tourist scenes through multimodal fusion

**DOI:** 10.3389/fnbot.2024.1504070

**Published:** 2025-01-10

**Authors:** Xuefan Zhou, Jiapeng Li, Yingzheng Li

**Affiliations:** ^1^College of Tourism Management, Guizhou University of Commerce, Guiyang, China; ^2^School of Computer Science and Technology, Guangdong University of Technology, Guangzhou, China; ^3^School of Business, Lingnan University, Hong Kong, China

**Keywords:** pedestrian detection, infrared and visible light, FusionU10 model, YOLOv10, AGUNet

## Abstract

With the rapid development of tourism, the concentration of visitor flows poses significant challenges for public safety management, especially in low-light and highly occluded environments, where existing pedestrian detection technologies often struggle to achieve satisfactory accuracy. Although infrared images perform well under low-light conditions, they lack color and detail, making them susceptible to background noise interference, particularly in complex outdoor environments where the similarity between heat sources and pedestrian features further reduces detection accuracy. To address these issues, this paper proposes the FusionU10 model, which combines information from both infrared and visible light images. The model first incorporates an Attention Gate mechanism (AGUNet) into an improved UNet architecture to focus on key features and generate pseudo-color images, followed by pedestrian detection using YOLOv10. During the prediction phase, the model optimizes the loss function with Complete Intersection over Union (CIoU), objectness loss (obj loss), and classification loss (cls loss), thereby enhancing the performance of the detection network and improving the quality and feature extraction capabilities of the pseudo-color images through a feedback mechanism. Experimental results demonstrate that FusionU10 significantly improves detection accuracy and robustness in complex scenes on the FLIR, M3FD, and LLVIP datasets, showing great potential for application in challenging environments.

## 1 Introduction

According to statistics from the World Tourism Organization, the number of international tourists has increased significantly over the past decade, and the total revenue of the tourism industry has steadily accounted for a growing proportion of global GDP (Bi et al., 2021). However, alongside the rapid development of tourism, several issues have emerged, particularly in popular tourist destinations and urban tourism centers. The high-density flow of tourists in these areas poses serious challenges to public safety and resource management. In overcrowded tourist attractions, congestion and disorder not only negatively impact the visitor experience but can also lead to a range of social issues, including stampedes, public security concerns, and the overuse of local infrastructure. These problems not only jeopardize the safety of tourists but also threaten the sustainable development of the destinations (Sperĺı, 2021; He et al., 2021). Therefore, accurate pedestrian detection technology is becoming increasingly essential. By leveraging advanced artificial intelligence and computer vision technologies, it is possible to monitor tourist flow in real-time, predict crowd behavior, and provide dynamic solutions for safety management and resource allocation (Chang et al., 2020). Especially in the event of emergencies or overcrowding, precise pedestrian detection can effectively reduce the risk of accidents, improve management efficiency, and support the healthy development of the tourism industry.

Despite the significant advancements in object detection technology, many challenges still remain. Firstly, under low-light conditions, traditional object detection algorithms often struggle to accurately identify pedestrians (Wei J. et al., 2023; Krishnendhu and Mohandas, 2023). In nighttime or poorly lit environments, pedestrian contours become blurred, and the loss of color and texture information leads to a substantial decrease in detection accuracy. Secondly, the issue of occlusion among pedestrians further complicates detection. In crowded areas, such as tourist attractions, pedestrians are often partially or completely obscured, making it difficult for existing detection models to effectively recognize those obstructed, thus affecting the overall detection performance (Bao et al., 2023; Gupta et al., 2023). Moreover, infrared images often lack rich visual cues, such as color and detailed information, which makes pedestrian detection based on infrared images more complex. Additionally, infrared images are prone to interference from background noise, especially in complex outdoor environments, where heat sources in the background may exhibit characteristics similar to those of pedestrians, further compromising detection accuracy.

In recent years, the YOLO (You Only Look Once) algorithm has made significant progress in the field of object detection, especially excelling in real-time applications such as pedestrian detection. Due to its high detection speed and accuracy, the YOLO model has been widely applied in intelligent tourism systems to monitor crowd movements in tourist destinations in real-time. However, despite its advancements, the YOLO algorithm still faces some critical challenges in practical deployment (Aboah et al., 2023). Firstly, the post-processing phase of the YOLO algorithm heavily relies on Non-Maximum Suppression (NMS) to eliminate overlapping detection boxes and ensure detection accuracy. This reliance makes it difficult to achieve true end-to-end deployment with YOLO (Wang et al., 2024; Wei H. et al., 2023). The introduction of NMS adds extra computational burden, leading to inference delays, which can affect real-time monitoring, especially in complex scenarios such as tourist sites that require quick response times. Secondly, the individual components of the YOLO model lack comprehensive and systematic optimization within the existing architecture, resulting in increased computational redundancy. When dealing with complex backgrounds and pedestrian occlusion in tourist attractions, some computational steps may be repeated unnecessarily, further reducing the system's response efficiency. These limitations can hinder the YOLO algorithm from achieving the expected real-time performance in large-scale pedestrian detection tasks in complex tourism environments (Wang et al., 2024).

Based on the limitations of existing object detection technologies, researchers are now exploring multimodal approaches that fuse infrared and visible light information to enhance pedestrian detection performance. Infrared images provide additional thermal information in low-light conditions, while visible light images offer rich detail and color cues. By combining these two types of information, pedestrian detection models can more accurately identify targets in complex scenarios, particularly in nighttime or heavily occluded situations, significantly improving detection robustness. This multimodal approach has become a key direction to address the shortcomings of traditional single-source detection methods. At the same time, the YOLO algorithm continues to evolve to meet the challenges of practical applications. The newly proposed YOLOv10 introduces a dual-assignment NMS-free training mechanism, eliminating the dependency on traditional Non-Maximum Suppression (NMS). This allows the model to avoid the additional computational overhead during inference, resulting in lower inference latency while maintaining competitive performance. Additionally, YOLOv10 adopts an efficiency-accuracy-driven model design strategy, optimizing the computational processes of its components, thereby significantly improving computational efficiency while maintaining high precision.

This paper proposes a model named FusionU10, which leverages the fusion of multimodal information from infrared and visible light to significantly improve the accuracy and robustness of pedestrian detection. The model generates pseudo-color images using an improved UNet network and integrates the YOLOv10 network for pedestrian detection. By merging multi-channel features from both infrared and visible light images, the generated pseudo-color images capture more detailed information, enhancing detection performance. Subsequently, the YOLOv10 network performs efficient object recognition on these images, ensuring the model maintains strong detection capabilities even in complex environments.

The main contributions of this paper are as follows:

- This paper designs the FusionU10 model, combining an improved UNet architecture with the YOLOv10 pedestrian detection network. Through the tight integration of pseudo-color image generation and object detection, the model achieves more accurate pedestrian detection in low-light and complex scenes.
- This paper proposes the AGUNet network, which introduces an AGUNet in the skip connections of the UNet architecture. This mechanism effectively filters redundant information and enhances focus on key features, enabling more precise detection of pedestrians, particularly in infrared images.
- This paper designs a joint loss function that integrates CIoU loss, object confidence loss, and classification loss. It not only optimizes the YOLOv10 detection network but also improves the quality of pseudo-color image generation and the effectiveness of feature extraction through a feedback mechanism, thereby comprehensively enhancing the model's performance and robustness.

The structure of the remaining sections of this paper is as follows: The second section covers related work, focusing on a review of the development of the YOLO algorithm and its application in pedestrian detection, as well as the latest advancements in multimodal fusion technology. The third section provides a detailed explanation of the proposed method, introducing the design and implementation details of the FusionU10 model. The fourth section presents the experiments, including a description of the datasets used, comparative experiments with state-of-the-art methods, and ablation studies. Finally, the conclusion section summarizes the research findings and suggests future research directions.

## 2 Related work

### 2.1 YOLO algorithm advancements in pedestrian detection

The YOLO algorithm has made significant progress in the field of pedestrian detection, with each version optimized and improved for different application scenarios and requirements (Soylu and Soylu, 2023). Firstly, YOLOv3 (Guillermo et al., 2023) introduced a multi-scale prediction mechanism, enabling detection on feature maps of various sizes. This allowed it to perform exceptionally well in detecting targets of different sizes, particularly in pedestrian detection, where YOLOv3 demonstrated strong detection capabilities for distant and small-sized targets. Additionally, YOLOv3 adopted Darknet-53 as its backbone network, enhancing the depth and breadth of feature extraction, which allowed it to maintain high detection accuracy even in complex backgrounds. Following this, YOLOv4 (Li et al., 2023) built upon YOLOv3 and introduced further improvements, striking a better balance between inference speed and detection accuracy. YOLOv4 incorporated the Mish activation function and the Cross Stage Partial (CSP) structure, significantly improving computational efficiency while reducing redundant calculations. Furthermore, YOLOv4 integrated Bag of Freebies (BoF) and Bag of Specials (BoS) techniques, such as data augmentation, DropBlock regularization, and the CIoU loss function. These enhancements greatly increased the model's generalization ability, making YOLOv4 particularly effective in crowded tourist attractions or densely populated environments, where it demonstrated excellent adaptability to complex lighting conditions and pedestrian occlusion. YOLOv5 (Chen, 2022) pushed forward with a lightweight design, significantly reducing computational resource requirements while maintaining detection accuracy. Through the use of automatic mixed data augmentation and an optimized loss function, YOLOv5 showed notable improvements in detecting small targets. Since pedestrian detection tasks often require high real-time performance, YOLOv5's lightweight design makes it well-suited for deployment on resource-constrained devices, such as drones and mobile surveillance systems, providing technical support for rapid response. Next, YOLOv6 (Soylu and Soylu, 2023) excelled in industrial applications by further optimizing inference speed and precision. With the introduction of the RepVGG module and more efficient training strategies, YOLOv6 performed more efficiently and stably when handling large-scale pedestrian detection tasks. In large-scale dynamic scenarios, such as tourist destinations, YOLOv6's optimized architecture delivered fast detection responses, greatly enhancing system monitoring capabilities. YOLOv7 (Zhao H. et al., 2023) made further breakthroughs in balancing speed and accuracy. It utilized the Extended Efficient Layer Aggregation Networks (ELAN) structure and gradient flow-based optimization, improving detection performance without significantly increasing computational complexity. Particularly when dealing with dense crowds and pedestrian occlusion, YOLOv7 demonstrated superior detection capabilities. Moreover, YOLOv7 included targeted optimizations for pedestrian detection across different resolutions, enabling more accurate identification of various targets and further improving detection robustness. YOLOv8 (Aboah et al., 2023) introduced adaptive architecture search technology, allowing the model to automatically adjust its network structure based on the requirements of the scene, thereby achieving higher detection accuracy in complex environments. In pedestrian detection tasks, YOLOv8 could adaptively handle complex backgrounds, lighting variations, and dense crowds, particularly excelling in addressing occlusion issues. Additionally, YOLOv8 employed a new loss function that optimized bounding box regression, making pedestrian detection results more precise and providing essential support for intelligent surveillance systems in tourist destinations. Finally, YOLOv9 (Bakirci and Bayraktar, 2024) brought several innovations, including a dual-assignment mechanism and an NMS-free training strategy, completely eliminating dependence on traditional Non-Maximum Suppression (NMS). This effectively reduced computational overhead during inference, lowering inference latency. YOLOv9 demonstrated excellent real-time performance in dense scenarios, especially in large-scale crowd monitoring in tourist destinations. Furthermore, YOLOv9 integrated PGI and a multi-functional GELAN architecture, further optimizing the computational flow of its components. This resulted in significant improvements in inference speed while maintaining high precision, making it one of the top choices for pedestrian detection today.

### 2.2 Multimodal fusion algorithms in pedestrian detection

Multimodal fusion algorithms have gained widespread attention in pedestrian detection tasks in recent years, by combining information from different modalities (such as infrared and visible light images) to enhance detection robustness and accuracy. Firstly, the weighted average fusion-based pedestrian detection algorithm is a relatively classic multimodal fusion method that improves pedestrian detection performance under different lighting conditions by performing weighted average fusion at the feature layer of infrared and visible light images (Zhao X. et al., 2023; Ning et al., 2023). Its advantage lies in its simplicity, effectively mitigating the limitations of a single modality in low-light conditions. However, its drawback is the lack of deep understanding of different modality information, and the fixed fusion strategy makes it less adaptable to complex environmental changes (Wei J. et al., 2023; Li et al., 2021; Chen et al., 2024). Secondly, the deep feature fusion-based multimodal pedestrian detection algorithm utilizes convolutional neural networks (CNN) to extract deep features from both infrared and visible light images, followed by fusion at later stages. This type of algorithm leverages the strong representational power of deep learning models, capturing richer semantic information from both modalities. Its advantage lies in effectively exploiting the complementary characteristics of different modalities, thus improving detection accuracy. However, due to the complexity of deep networks, this method involves high computational costs and its efficiency in real-time applications requires further optimization (Fu et al., 2024). Thirdly, the attention mechanism-based multimodal fusion algorithm introduces an attention mechanism in pedestrian detection to dynamically assign weights to different modalities, enhancing focus on important regions. The strength of this approach lies in its ability to adaptively adjust the fusion ratio of infrared and visible light information based on the complexity of the scene, effectively handling occlusions and complex backgrounds. However, its limitation is that training the attention mechanism relies heavily on large amounts of labeled data, and in some extreme scenarios, it may cause modality imbalance, affecting detection performance. Fourthly, the generative adversarial network (GAN)-based multimodal fusion algorithm proposes a method of generating virtual modality images through GANs and fusing them with actual modalities. This method effectively enhances the diversity of modality information, especially in cases where infrared or visible light data is missing, by generating the missing modality information using the generator, enabling more robust pedestrian detection. Its advantage is improving detection performance under extreme conditions, but its drawback lies in the high training demands of GAN models, which may lead to mode collapse or poor-quality generated images (Jing et al., 2023). Finally, the self-supervised learning-based multimodal fusion pedestrian detection algorithm uses self-supervised learning to enhance the model's understanding of multimodal data in situations where large amounts of labeled data are lacking. This algorithm performs well in data-scarce environments by pretraining on unlabeled infrared and visible light images, thereby improving the model's generalization ability. However, its limitation is the high complexity of designing self-supervised tasks, and the pretraining process can be time-consuming (Wu et al., 2024; Li and Shen, 2023). In summary, multimodal fusion algorithms excel in pedestrian detection tasks under complex conditions by combining the advantages of different modalities. However, while these algorithms improve detection accuracy, they also face challenges in terms of computational cost, training complexity, and adaptability.

In recent years, multi-modal fusion algorithms have garnered significant attention in pedestrian detection tasks, enhancing robustness and accuracy by combining information from different modalities, such as infrared and visible light images. The main types of methods are as follows: First, weighted average fusion-based pedestrian detection algorithms are among the earliest multi-modal fusion approaches, which enhance detection in various lighting conditions by averaging the infrared and visible light images at the feature layer. These methods are computationally simple and effective for low-light environments, but they employ a fixed fusion strategy that lacks deeper understanding of modality information, making them less adaptable to complex environments (Zhao X. et al., 2023). Secondly, deep feature fusion-based multi-modal pedestrian detection algorithms use convolutional neural networks (CNNs) to extract deep features from infrared and visible light images separately, then fuse these at later stages. Leveraging the representational power of deep learning, these methods effectively capture the complementary characteristics between modalities, boosting detection accuracy. However, their complexity results in high computational costs, and real-time performance needs improvement (Wei J. et al., 2023; Li et al., 2021). Attention mechanism-based multi-modal fusion algorithms introduce dynamic weight assignment to emphasize key regions during pedestrian detection. Through attention mechanisms, these algorithms adaptively adjust the fusion ratio of infrared and visible light information, improving robustness against occlusions and complex backgrounds. However, they heavily rely on large datasets for training and may experience modality imbalance in extreme scenarios, affecting detection quality (Fu et al., 2024; Zhao Z. et al., 2023). To further address image degradation, Degradation-Robust Multi-modality image Fusion (DRMF) (Tang et al., 2024) leverages the generative power of diffusion models to counter low-light, low-resolution, and noisy conditions, making it especially suited for complex infrared-visible and medical image fusion tasks. In the realm of target-aware fusion, the Target-Aware Dual Adversarial Learning (TarDAL) (Liu et al., 2022) network employs a dual-discriminator structure to extract complementary information between modalities, retaining structural details from infrared images and texture from visible light, thus enhancing fusion quality and detection accuracy. Additionally, Illumination-Aware Infrared and Visible Image Fusion Network (IAIFNet) (Yang et al., 2024) is designed for low-light environments with an illumination enhancement module, combined with a salient target-aware module and an adaptive differential fusion module. This configuration enables brightness-sensitive high-quality fusion and significantly improves detection performance in low-light scenarios. Within the GAN framework, GAN-based multi-modal fusion algorithms generate virtual modality images and fuse them with real images to enhance modality diversity. This approach excels when either infrared or visible light images are missing, although it requires intensive training, with risks of mode collapse or lower image quality (Jing et al., 2023; Wang et al., 2023). Finally, self-supervised learning-based multi-modal fusion algorithms enhance model understanding of multi-modal data in the absence of large annotated datasets. These methods perform well in data-scarce environments, utilizing unlabeled infrared and visible light images for pretraining to boost model generalization. However, the pretraining process is time-intensive, and designing effective tasks is complex (Wu et al., 2024; Li and Shen, 2023). In summary, multi-modal fusion algorithms excel in pedestrian detection under challenging conditions by combining the strengths of different modalities. However, despite their improved accuracy, these algorithms face challenges related to computational cost, training complexity, and adaptability.

## 3 Method

### 3.1 FusionU10

Our model FusionU10 consists of two key components: (1) a pseudo-color image generation network based on an improved UNet architecture, and (2) a pedestrian detection main network based on YOLOv10 (Wang et al., 2024). These two networks are closely integrated to achieve efficient pedestrian detection tasks. First, the input network utilizes infrared and visible light image data, extracting three-channel and four-channel features respectively. After selecting and fusing different spectral information through a feature selection module, the data is input into the AGUNet network. The improved UNet adopts an encoder-decoder architecture, where the encoder extracts multi-scale features through convolution and downsampling operations, and the decoder reconstructs feature maps through deconvolution and upsampling operations. These are then fused with the encoder feature maps, ultimately producing a three-channel pseudo-color image. Next, the pseudo-color image is passed through the YOLOv10 pedestrian detection main network for object detection. The YOLOv10 structure consists of three modules: Backbone, Neck, and Head. The Backbone is responsible for feature extraction, the Neck handles feature fusion and enhancement, and the Head performs object prediction. The loss function during the prediction phase consists of three parts: box loss, object loss, and class loss. This loss function not only optimizes the detection main network but also feeds back to improve the UNet, thereby enhancing the quality of the pseudo-color image and the feature extraction capability.

**Figure 1:**
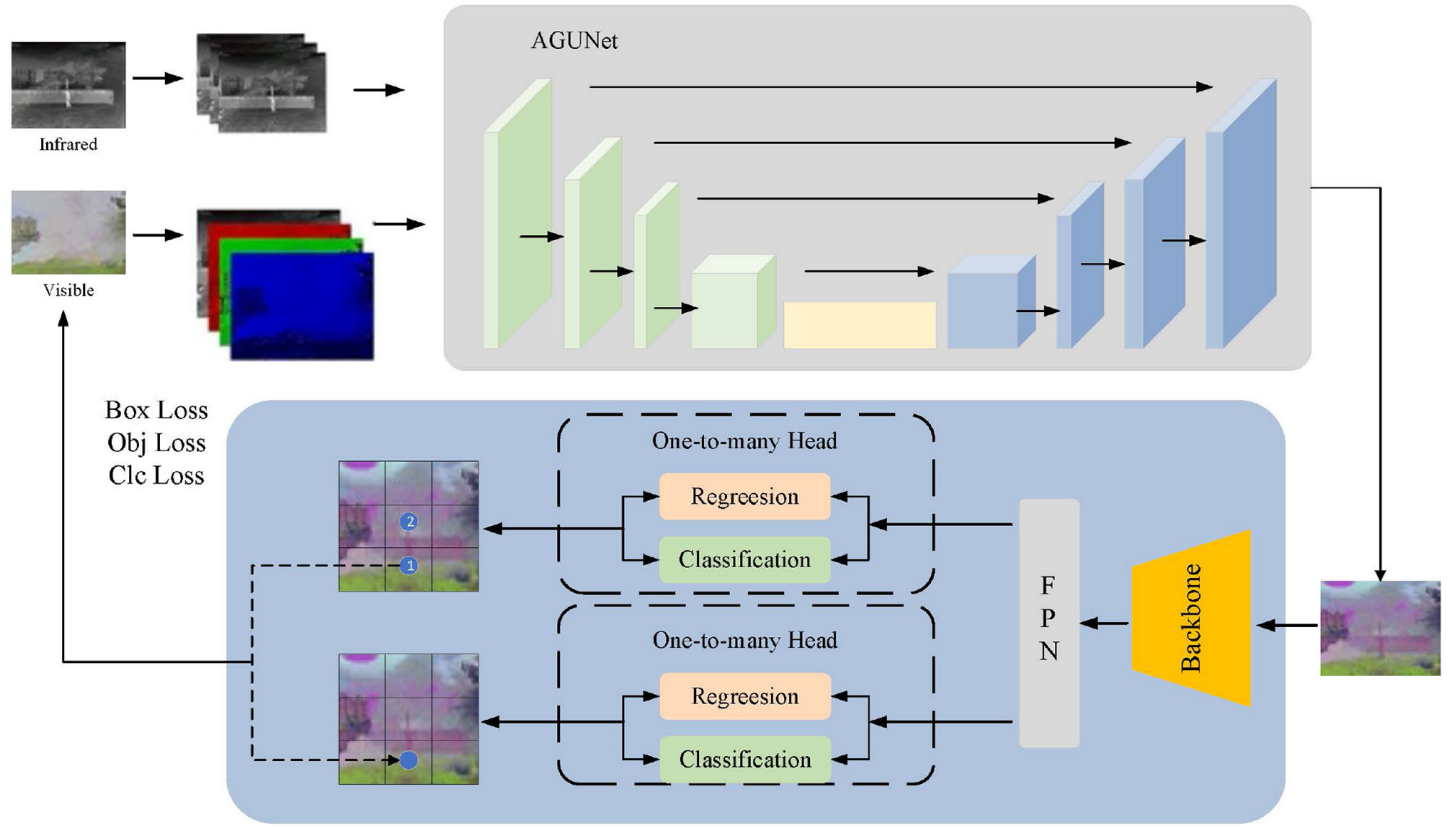
The overall network structure of FusionU10. It mainly includes the pseudo-color image generation network of the AGUNet architecture and the main network for pedestrian detection using YOLOv10.

The network structure of FusionU10 is shown in Figure 1, which tightly integrates the pseudo-color image generation with YOLOv10 pedestrian detection. By jointly optimizing both components, the model significantly improves the accuracy and robustness of pedestrian detection. By effectively extracting and fusing features from different spectral information, FusionU10 demonstrates excellent performance in object detection tasks in complex environments.

### 3.2 AGUNet

AGUNet enhances the traditional UNet model by incorporating Attention Gates (AG) to more effectively focus on significant features in the input images while ignoring irrelevant background areas. The core innovation lies in the use of AG for feature selection in the skip connections, where Gating Signals are employed to integrate multi-scale contextual information, ensuring the preservation of key features and filtering out redundant information. This mechanism is particularly crucial for infrared pedestrian detection tasks, as infrared images often contain a large amount of irrelevant data.

**Figure 2:**
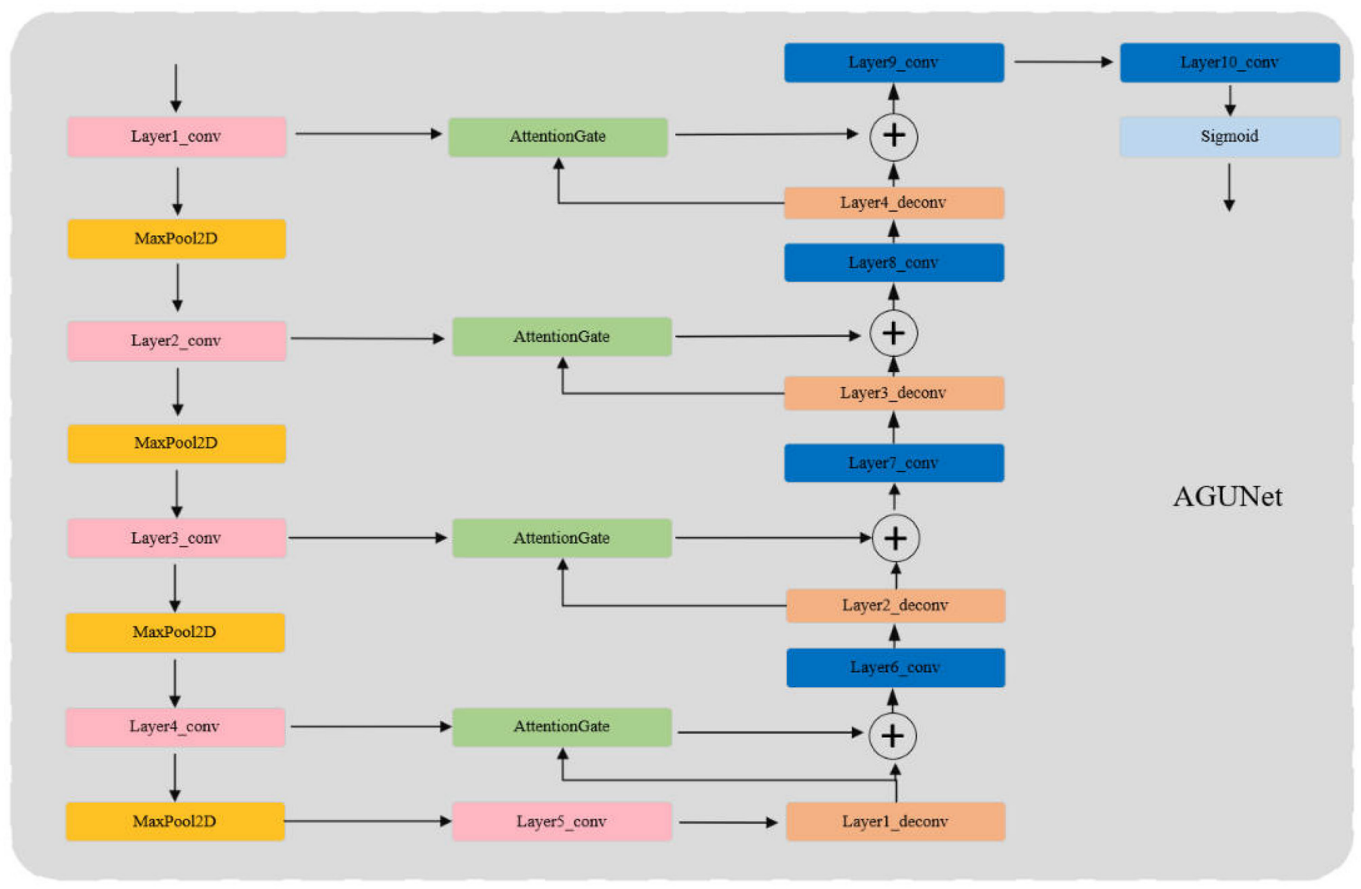
AGUNet network architecture.

**Figure 3:**
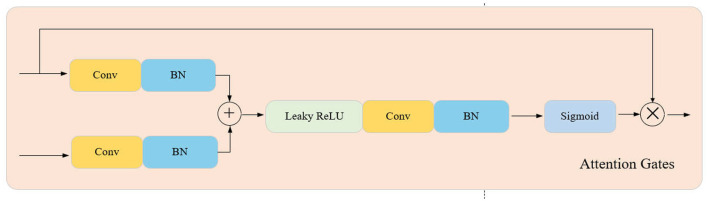
Attention gates network architecture.

As shown in Figure 2, the structure of AGUNet consists of an encoder and a decoder, with AG inserted into the skip connections between each layer of the encoder and decoder. The encoder progressively downsamples the input through multiple convolutional and pooling layers to extract multi-scale features. In each layer, AG selectively filters the features propagated through the skip connections, ensuring that only features beneficial to the detection task are transmitted to the decoder. Features first pass through AG, where their importance is evaluated by the Gating Signal, before being sent to the corresponding decoder layer for upsampling and feature fusion. The decoder, symmetrical to the encoder, gradually restores the spatial resolution of the image through deconvolution layers, ultimately outputting a three-channel pseudo-color image via the Sigmoid activation function. During the decoding process, AG not only enhances significant features but also ensures effective fusion of features at different scales. Moreover, the attention coefficients (α) calculated in AG are used to scale the input features, adaptively highlighting key regions and further improving feature representation, significantly enhancing the network's ability to capture fine details. The lightweight design of AGUNet is achieved by removing two encoder and decoder layers from the traditional UNet, which reduces computational complexity and increases inference speed, making it suitable for real-time pedestrian detection tasks.The structure of the Attention Gate network is shown in Figure 3.

### 3.3 Loss function

In the design of the AGUNet loss function, we start with the traditional UNet loss function. The traditional UNet primarily uses pixel-wise cross-entropy loss to measure the difference between the predicted segmentation mask and the ground truth mask. The basic formula is:


(1) $$\begin{array}{l} L_{\text{CE}}=-\frac{1}{N}\overset{N}{\underset{i=1}{\sum }}\left(y_{i}\log\left({ŷ}_{i}\right)+\left(1-y_{i}\right)\log\left(1-{ŷ}_{i}\right)\right) \end{array}$$

where *y*_*i*_ is the ground truth label, ŷ_*i*_ is the predicted probability, and *N* is the total number of pixels in the image. The cross-entropy loss calculates the error for each pixel and takes the average, ensuring that the model accurately predicts the classification of each pixel.

In our proposed AGUNet, we further combine the YOLOv10 pedestrian detection task into a joint loss function. This combined loss function integrates three parts: CIoU loss for optimizing bounding box predictions, objectness loss for determining object presence, and classification loss. In this context, *S*^2^ denotes the grid size of the feature map, and *B* represents the number of bounding boxes predicted per grid cell. The combined loss function is defined as follows:


(2) $$\begin{array}{l} ℒ(t_{p},t_{gt})=\sum_{k=0}^{K}[\alpha_{k}^{\text{balance}}\alpha_{\text{box}}\sum_{i=0}^{S^{2}}\sum_{j=0}^{B}1_{\left\{kij\right\}}^{\text{obj}}{ℒ}_{\text{CIoU}}\\ \;+\alpha_{\text{obj}}\sum_{i=0}^{S^{2}}\sum_{j=0}^{B}1_{\left\{kij\right\}}^{\text{obj}}{ℒ}_{\text{obj}}+\alpha_{\text{cls}}\sum_{i=0}^{S^{2}}\sum_{j=0}^{B}1_{\left\{kij\right\}}^{\text{obj}}{ℒ}_{\text{cls}}] \end{array}$$

This combined loss function integrates the CIoU loss $L_{\text{CIoU}}$ (Equation 3), objectness loss $L_{\text{obj}}$ (Equation 4), and classification loss $L_{\text{cls}}$ (Equation 5), which are balanced by their respective coefficients α_box_, α_obj_, and α_cls_. This ensures that the loss function optimizes both pseudo-color image generation and pedestrian detection accuracy, improving the model's robustness.

The formula for CIoU Loss is used to optimize the prediction of bounding boxes by considering the overlap, center point distance, and aspect ratio to improve detection accuracy. The specific formula is as follows:


(3) $$\begin{array}{l} L_{\text{CIoU}}=1-\text{IoU}+\frac{\rho^{2}\left(b,b_{gt}\right)}{c^{2}}+\alpha v\left(1-\text{IoU}\right)+v \end{array}$$

where IoU is the Intersection over Union, $\rho^{2}\left(b,b_{gt}\right)$ the center distance, *c*^2^ the enclosing box diagonal, α the aspect ratio parameter, and *v* the aspect ratio consistency.

Next, the object loss *L*_obj_ is used to penalize the network for incorrect predictions of the presence of an object. The specific formula is:


(4) $$\begin{array}{l} L_{\text{obj}}=-\overset{S^{2}}{\underset{i=1}{\sum }}\overset{B}{\underset{j=1}{\sum }}{\text{1}}_{\left\{ij\right\}}^{\text{obj}}\log\left(p_{\text{obj}}\right) \end{array}$$

where *p*_obj_ represents the probability predicted by the network that an object exists in the position, and ${\text{1}}_{\left\{ij\right\}}^{\text{obj}}$ is an indicator function that equals 1 when an object is indeed present in the grid cell.

Finally, the classification loss *L*_cls_ is used to optimize the network's prediction of the object's class. The formula is:


(5) $$\begin{array}{l} L_{\text{cls}}=-\overset{S^{2}}{\underset{i=1}{\sum }}\overset{B}{\underset{j=1}{\sum }}{\text{1}}_{\left\{ij\right\}}^{\text{obj}}\overset{C}{\underset{c=1}{\sum }}\left[p_{ijc}\log\left({\hat{p}}_{ijc}\right)\right] \end{array}$$

where *p*_*ijc*_ is the true probability distribution of the class, ${\hat{p}}_{ijc}$ is the class probability distribution predicted by the network, and *C* represents the total number of classes.

## 4 Experiments

### 4.1 Dataset

This paper utilizes three main datasets for experimentation and validation: the FLIR (Li et al., 2021), M3FD (Liu et al., 2022), and LLVIP (Jia et al., 2021) datasets. These datasets provide a substantial amount of infrared and visible light images, covering pedestrian detection tasks under various scenes and environmental conditions, particularly in challenging lighting scenarios.

FLIR Dataset: The FLIR dataset contains 14,452 infrared images, including 10,228 images extracted from multiple short videos, and an additional 4,224 images from a 144-second long video. The image resolution is 640x512, primarily used to evaluate the performance of pedestrian detection algorithms in low-light or nighttime environments. The FLIR dataset offers diversity in terms of weather and temperature conditions, and the annotations include bounding boxes for pedestrians and vehicles, providing rich data for training and evaluation in object detection tasks.

M3FD Dataset: The M3FD dataset is specifically designed for multimodal pedestrian detection tasks and contains 4,200 pairs of synchronized visible light and infrared images. Additionally, the dataset provides 300 pairs of fused images that combine infrared and visible light information, offering valuable data for validating multimodal fusion algorithms. The M3FD dataset captures scenes under complex backgrounds and lighting conditions, making it suitable for evaluating the performance of fusion networks when dealing with occlusions, low contrast, and varying lighting environments.

LLVIP Dataset: The LLVIP dataset includes 30,976 images, comprising 15,488 pairs of visible light and infrared images, mainly used to evaluate object detection and recognition tasks in low-light conditions. The images in this dataset are captured from different urban street scenes, covering multiple object categories such as pedestrians and vehicles, and are specifically optimized for extreme nighttime environments with low illumination. In addition to providing precise bounding box annotations, the dataset contains rich scene information, such as dense pedestrian areas and open spaces, making it an essential resource for multimodal pedestrian detection experiments.

### 4.2 Experimental setup

#### 4.2.1 Experimental environment

The experiments in this study were conducted in the following hardware and software environment. The hardware platform consists of a server equipped with an Intel Xeon Gold 6226R @ 2.90 GHz processor, 256GB DDR4 memory, and an NVIDIA Tesla V100 GPU (32GB) to accelerate the training and inference of deep learning models. The operating system used was Ubuntu 20.04 LTS (64-bit), ensuring compatibility with deep learning frameworks. In terms of software, the development was carried out using Python 3.9. The deep learning models were implemented using the PyTorch 1.10.1 framework, which is widely used in research areas such as computer vision and natural language processing. CUDA 11.3 was employed to accelerate GPU computing, while cuDNN 8.2 was utilized to optimize the performance of convolutional neural networks. Additionally, Anaconda 2021.11 was used to manage project dependencies, ensuring compatibility between different libraries. To guarantee the reproducibility of the experimental results, all experiments were run multiple times under the same environment, with relevant log data recorded for each run. The above configuration ensured efficiency and stability in the training, testing, and validation of the models.

#### 4.2.2 Experimental details

To ensure the generalization ability of the model, the FLIR, M3FD, and LLVIP datasets were divided according to specific ratios, constructing training and test sets to optimize training and evaluate performance. As shown in Table 1, the FLIR dataset contains 8,862 annotated infrared images for training, covering various weather and lighting conditions, and provides 1,366 images for testing, evaluating the model's performance in low-light and complex scenarios. The M3FD dataset is split in an 8:2 ratio, with 3,360 pairs of visible light and infrared images in the training set and 840 pairs in the test set, used to verify detection performance under multimodal fusion. The LLVIP dataset retains its original split, with 12,025 image pairs for training and 3,463 pairs for testing, focusing on evaluating the model's detection performance under low-light conditions.

**Table 1:** Dataset splits for training and testing.

**Dataset**	**Training set**	**Test set**
FLIR	8,862	1,366
M3FD	3,360	840
LLVIP	12,025	3,463

During the training process of FusionU10, the weights of the UNet are initialized randomly, while YOLOv10 loads pre-trained weights on visible light (YOLOv10s.pt). The “Frozen-backbone Training” strategy refers to freezing the backbone of YOLOv10 during training to maintain stability in feature extraction. In the “Fine-tuning” phase, the entire feature extraction component, including both the UNet and YOLOv10 backbone, is frozen to ensure efficient multimodal feature fusion and stable model optimization.

#### 4.2.3 Parameter settings

During the network training phase, we conducted initial parameter settings, including selecting appropriate batch size, number of epochs, learning rate, and optimizer type. The specific settings are shown in Table 2.

**Table 2:** Training parameters.

**Parameter**	**Value**
Learning rate	0.001
Batch size	32
Weight decay	0.0005
Epochs	300
Layers	225
Optimizer	Adam

### 4.3 Evaluation metrics

This paper employs multiple evaluation metrics to assess the performance of the pedestrian detection model, including IOU (Intersection over Union), F1 score, FPS (Frames Per Second), and GFLOP (Giga Floating Point Operations Per Second). These metrics evaluate the model from different aspects, such as accuracy, speed, and computational efficiency.

IOU is a commonly used metric in object detection, which measures the overlap between the predicted bounding box and the ground truth box. The formula is defined as follows:


(6) $$\begin{array}{l} IOU=\frac{Area\left(B_{pred}\cap B_{gt}\right)}{Area\left(B_{pred}\cup B_{gt}\right)} \end{array}$$

Where *B*_*pred*_ represents the predicted bounding box, *B*_*gt*_ represents the ground truth bounding box, ∩ denotes the intersection area, and ∪ denotes the union area of the two boxes. A detection is considered correct if the IOU value exceeds a certain threshold. In this paper, the IOU threshold is set to 0.5, meaning a detection is considered accurate when *IOU*≥0.5.

The F1 score is a metric that balances Precision and Recall. It is defined by the following formula:


(7) $$\begin{array}{l} F1=2\times \frac{Precision\times Recall}{Precision+Recall} \end{array}$$

### 4.4 Results analysis

As shown in Table 3, we compared the performance of different network architectures, input types, and training strategies in pedestrian detection tasks across the FLIR, M3FD, and LLVIP datasets. Analysis of the improvements in mean Average Precision (mAP) across different datasets indicates that the FusionU10 model achieves significant improvements in detection accuracy by incorporating multimodal fusion technology and optimized training strategies.

**Table 3:** Pedestrian detection results across FLIR, M3FD, and LLVIP datasets using different network architectures, input types, and training strategies.

**Dataset**	**Network architecture**	**Input type**	**Training strategy**	**Pedestrian**	**mAP@0.5**	**mAP@0.5–0.95**
FLIR	YOLOv5 (Chen, 2022)	Infrared	Full training	0.892	0.835	0.452
		YOLOv8 (Aboah et al., 2023)	Infrared	Full training	0.872	0.845	0.472
		YOLOv9 (Bakirci and Bayraktar, 2024)	Infrared	Full training	0.869	0.831	0.456
		YOLOv10 (Wang et al., 2024)	Infrared	Full training	0.878	0.843	0.467
		FusionU10	Infrared	Frozen-backbone training+Fine tuning	**0.899**	**0.857**	**0.497**
M3FD	YOLOv5 (Chen, 2022)	Infrared	Full training	0.864	0.818	0.528
		YOLOv5 (Chen, 2022)	Visible	Full training	0.771	0.866	0.570
		YOLOv8 (Aboah et al., 2023)	Infrared	Full training	0.900	0.869	0.604
		YOLOV8 (Aboah et al., 2023)	Visible	Full training	0.803	0.899	0.628
		YOLOv9 (Bakirci and Bayraktar, 2024)	Infrared+visible	Full training	0.818	0.893	0.609
		YOLOv10 (Wang et al., 2024)	Infrared+visible	Full training	0.880	0.899	0.623
		FusionU10	Infrared+visible	Frozen-backbone training+Fine tuning	**0.915**	**0.901**	**0.668**
LLVIP	YOLOv5 (Chen, 2022)	Infrared	Full training	0.952	0.632	0.651
		YOLOv5 (Chen, 2022)	Visible	Full training	0.834	0.436	0.430
		YOLOv8 (Aboah et al., 2023)	Infrared	Full training	0.971	0.732	0.548
		YOLOv8 (Aboah et al., 2023)	Visible	Full training	0.925	0.568	0.468
		YOLOv9 (Bakirci and Bayraktar, 2024)	Infrared+visible	Full training	0.975	0.696	0.551
		YOLOv10 (Wang et al., 2024)	Infrared+visible	Full training	0.974	0.697	0.697
		FusionU10	Infrared+visible	Frozen-backbone training+Fine tuning	**0.985**	**0.768**	**0.701**

Bold indicates the optimal result.

First, on the FLIR dataset, the mAP@0.5–0.95 of FusionU10 increased by 6.4% compared to YOLOv10. This improvement demonstrates that the training strategy of freezing the backbone and performing fine-tuning helps more effectively utilize multimodal fusion features, enhancing the model's accuracy in detecting small objects and in complex scenarios. Compared to YOLOv5 and YOLOv8, the mAP@0.5–0.95 of FusionU10 increased by 9.9% and 5.3%, respectively, further confirming its advantage in infrared image processing, enabled by the enhanced AGUNet architecture, which allows for more efficient extraction of key features under low-light conditions.

In the M3FD dataset, FusionU10 achieved an mAP@0.5–0.95 of 0.668, a 7.2% improvement over YOLOv10. Under multimodal input (infrared + visible light), FusionU10 further demonstrated its superior feature fusion capability, achieving a 6.4% improvement over YOLOv8, which used visible light input only. This result indicates that the multimodal fusion strategy effectively mitigates the impact of complex backgrounds and occlusions on model performance, significantly enhancing the model's robustness and detection accuracy.

On the LLVIP dataset, FusionU10 continued to exhibit the best performance, with an mAP@0.5–0.95 of 0.701. Although the improvement over YOLOv10 was only 0.4%, FusionU10 demonstrated greater stability and generalization in pedestrian detection tasks under low-light conditions. The strategy of freezing the backbone and fine-tuning enabled FusionU10 to make full use of the fused multimodal features, maintaining high detection accuracy in complex environments.

In summary, the FusionU10 model exhibits significant performance advantages by combining multimodal information fusion and optimized training strategies, particularly in challenging scenarios such as occlusion and low-light conditions. The experimental results validate the effectiveness and applicability of this model in improving detection accuracy, enhancing robustness, and adapting to complex detection tasks.

### 4.5 Performance comparison of different UNet architectures in pedestrian detection

In the ablation experiments of this paper, we compared the pedestrian detection performance of different UNet architectures on the M3FD and LLVIP datasets, as shown in Table 4. By integrating an Attention Gate into the AGUNet network, the detection performance was significantly improved, especially in terms of multimodal information fusion. Firstly, in the M3FD dataset, FusionU10 achieved an mAP@0.5–0.95 of 0.668, which represents an approximately 18% improvement compared to the 0.565 of the standard UNet+YOLOv10. This improvement indicates that the attention mechanism can effectively filter out irrelevant information and enhance the focus on key features, thus improving the accuracy of pedestrian detection. Compared to other improved UNet architectures, such as Inception U-Net and Dense U-Net, AGUNet showed a small difference in mAP@0.5, but it achieved the best performance in mAP@0.5–0.95, which is an indicator of overall performance across different thresholds. This suggests that AGUNet not only performs well under a single threshold but also demonstrates stronger robustness in more challenging multi-threshold detection tasks. In the LLVIP dataset, FusionU10 achieved an mAP@0.5–0.95 of 0.701, an improvement of approximately 8.3% over the 0.647 of standard UNet+YOLOv10. Although the difference in mAP@0.5 between AGUNet and Inception U-Net+YOLOv10 is not large, AGUNet still excels in overall performance.

**Table 4:** Pedestrian detection results using different UNet architectures on M3FD and LLVIP datasets.

**Network architecture**	**M3FD series**			**Low-income nations IP bureau**		
	**Avg mAP@0.5**	**mAP@0.5–0.95**	**F1 score**	**Avg mAP@0.5**	**mAP@0.5–0.95**	**F1 score**
UNet + YOLOv10; Azad et al. (2024)	0.846	0.565	0.84	0.960	0.647	0.93
Attention U-Net+YOLOv10; Oktay et al. (2018)	0.889	0.605	0.87	0.925	0.543	0.89
Inception U-Net + YOLOv10; Zhang et al. (2020)	0.899	0.623	0.89	**0.974**	0.697	**0.94**
Dense U-net + YOLOv10; Wang et al. (2019)	0.899	0.625	0.88	0.969	0.677	0.93
Residual U-Net + YOLOv10; Zhang et al. (2018)	0.895	0.625	0.88	0.972	0.673	0.94
AGUNet + YOLOv10	**0.901**	**0.668**	**0.89**	0.768	**0.701**	0.81

Bold indicates the optimal result.

**Figure 4:**
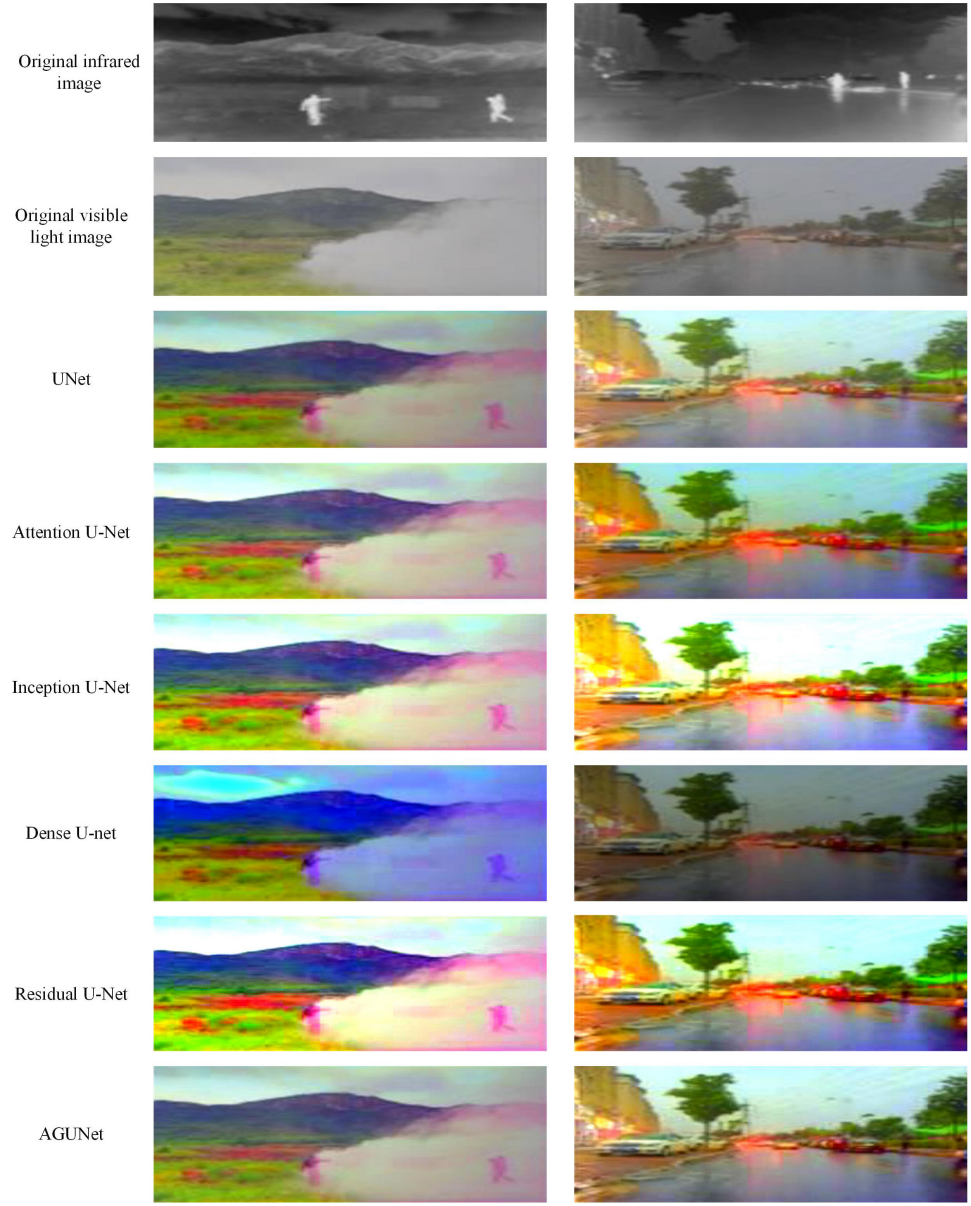
The output comparison images of different UNet architecture in M3FD dataset.

Figure 4 shows the output comparison images of different UNet architectures on the M3FD dataset. From the images, it can be seen that the strategies using the UNet architecture significantly enhance the detection results for pedestrian targets, especially in complex scenarios. AGUNet is able to better extract detailed features of pedestrians, demonstrating stronger robustness and precision. In contrast, the standard UNet is more susceptible to background noise, resulting in incomplete detection of some pedestrian targets. AGUNet, by introducing an attention mechanism, effectively suppresses the impact of irrelevant background information, making the pedestrian targets more prominent and the detection results more accurate. Overall, these results show that AGUNet not only maintains high detection accuracy under complex lighting conditions but also enhances robustness and accuracy in environments with heavy occlusion and noise through the attention mechanism, providing a new solution for pedestrian detection in multimodal information fusion scenarios.

#### 4.5.1 Performance comparison of different YOLO architectures in pedestrian detection

In this ablation study, we mainly compared the performance of different YOLO architectures in pedestrian detection tasks and conducted detailed experimental analysis using the FLIR, M3FD, and LLVIP datasets.

**Figure 5:**
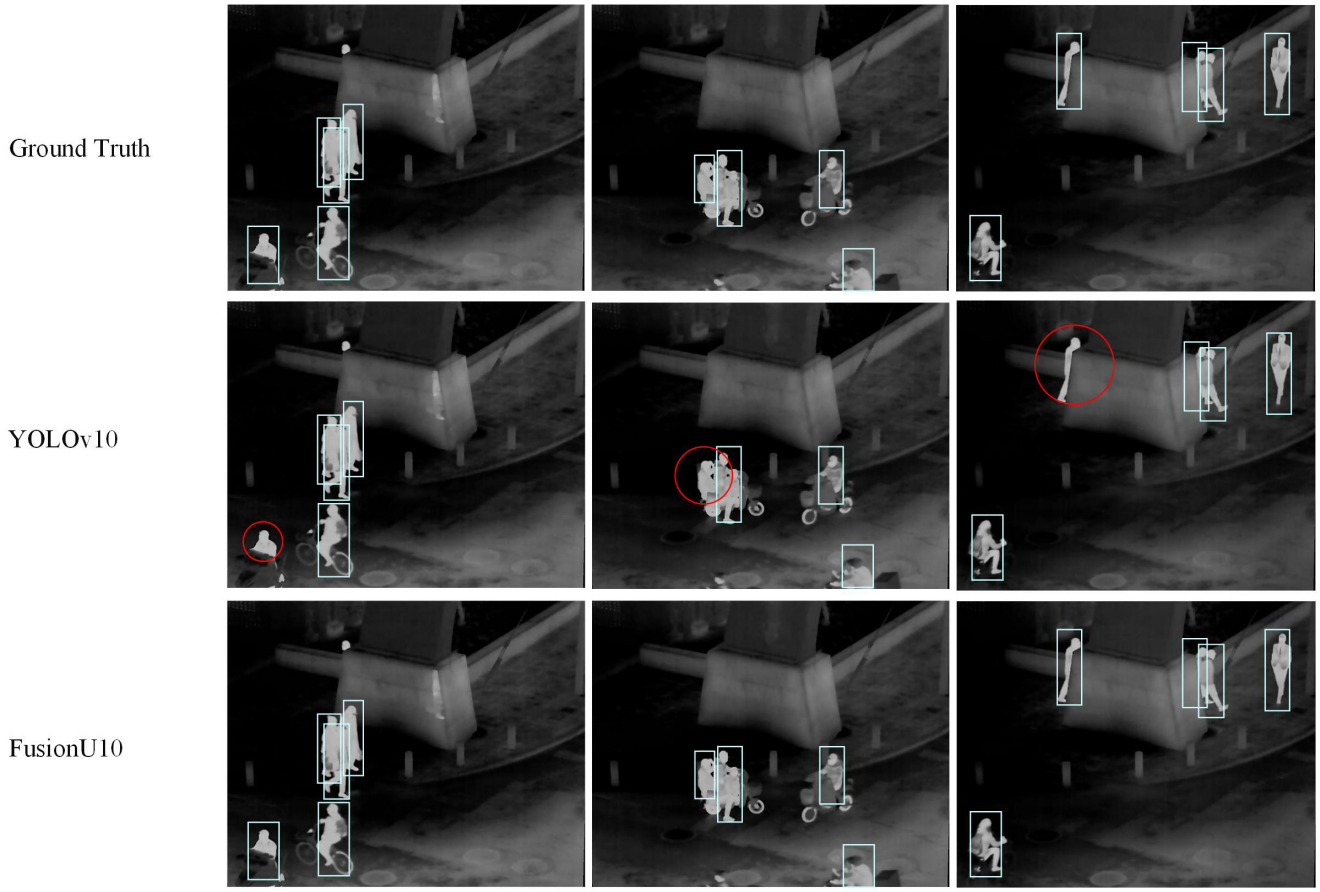
Pedestrian detection results on the FLIR dataset. The red circles represent incorrect detection information.

**Figure 6:**
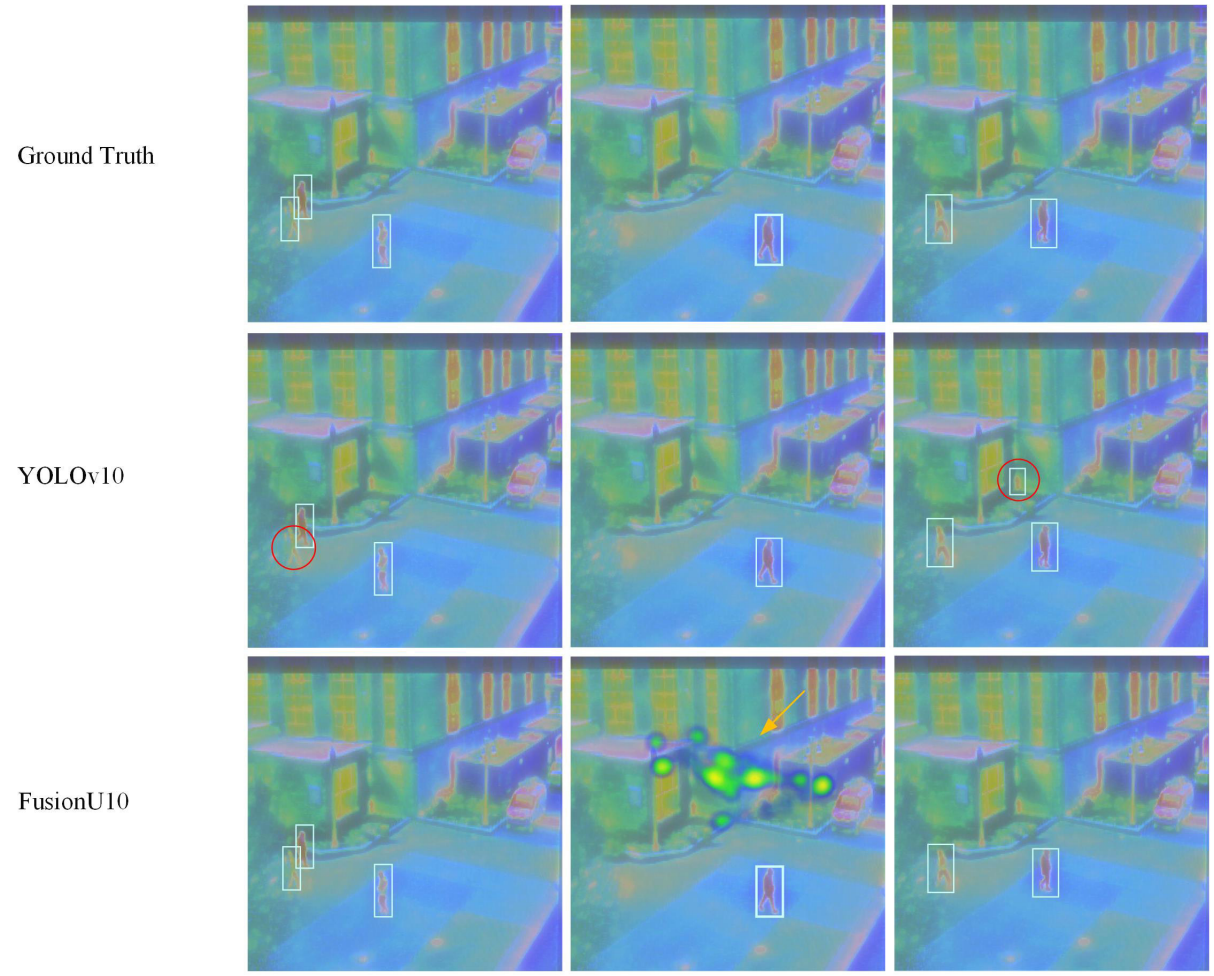
Pedestrian detection results on the M3FD dataset. The red circles represent incorrect detection information. The yellow arrows indicate that the UNet post-processing may produce certain areas of artifacts.

Firstly, Table 5 shows the results on the FLIR dataset. FusionU10, through frozen-backbone training and fine-tuning, achieved the highest mAP@0.5 and mAP@0.5–0.95, reaching 0.899 and 0.497, respectively. This represents a significant improvement over YOLOv5, YOLOv8, and YOLOv9, with the greatest increase being 17.5% in mAP@0.5–0.95. This indicates that YOLOv10, combined with AGUNet's multimodal information fusion and optimized training strategy, excels in pedestrian detection on infrared images. Figure 5 shows a comparison between our model and the original YOLOv10 in pedestrian detection, clearly illustrating that our model performs better in cases of occlusion and small objects. Especially in complex scenarios, FusionU10 can effectively handle situations where pedestrians are partially occluded or appear at a distance, while still maintaining high detection accuracy. In contrast, the original YOLOv10 shows instances of missed or false detections in similar cases. This further validates that after incorporating the attention mechanism, AGUNet can effectively enhance the capture of key features and reduce background noise interference, thus improving robustness across various scenarios. Figure 6 presents a comparison of pedestrian detection between our model and YOLOv10 on the M3FD dataset, showing that our model has a lower false positive rate. Additionally, the UNet post-processing introduces some noise in certain regions due to the limited contribution of these regions in the RGBT four-channel images to detection. As a result, the detection loss weights in these areas are difficult to adjust. However, this has minimal impact on the overall pedestrian detection performance of the model.

**Table 5:** The pedestrian detection results on FLIR dataset.

**Network architecture**	**Training strategy**	**Pedestrian mAP@0.5**	**mAP@0.5–0.95**	**F1 score**
AGUNet+YOLOv5	Frozen-backbone training	0.867	0.422	0.80
AGUNet+YOLOv8	Frozen-backbone training	0.791	0.435	0.71
AGUNet+YOLOv9	Frozen-backbone training	0.868	0.431	0.80
FusionU10	Frozen-backbone training+Fine tuning	**0.899**	**0.497**	**0.81**

Bold indicates the optimal result.

Next, on the M3FD dataset shown in Table 6, FusionU10 again achieved the best performance, with an mAP@0.5–0.95 of 0.668, which is a 22.2% improvement over YOLOv5, and also shows significant improvements over YOLOv8 and YOLOv9. This demonstrates that YOLOv10, with its optimized training strategy, better captures pedestrian targets and improves detection accuracy in complex backgrounds and multimodal data. Additionally, FusionU10 achieved an F1 score of 0.89, proving its reliability and accuracy in real detection tasks.

**Table 6:** The pedestrian detection results on M3FD dataset.

**Network architecture**	**Training strategy**	**Pedestrian mAP@0.5**	**mAP@0.5–0.95**	**F1 score**
AGUNet+YOLOv5	Frozen-backbone training	0.858	0.546	0.87
AGUNet+YOLOv8	Frozen-backbone training	0.801	0.589	0.82
AGUNet+YOLOv9	Frozen-backbone training	0.818	0.593	0.85
FusionU10	Frozen-backbone training+Fine tuning	**0.901**	**0.668**	**0.89**

Bold indicates the optimal result.

Finally, Table 7 presents the results on the LLVIP dataset. Although FusionU10 had a slightly lower mAP@0.5 compared to YOLOv9, it still achieved the best mAP@0.5–0.95, reaching 0.701, a 0.6% improvement over YOLOv9. This shows that the combination of AGUNet and YOLOv10 provides more robust detection performance in low-light and highly occluded scenarios.

**Table 7:** The pedestrian detection results on LLVIP dataset.

**Network architecture**	**Training strategy**	**mAP@0.5**	**mAP@0.5–0.95**	**F1 score**
AGUNet+YOLOv5	Frozen-backbone Training	0.655	0.647	0.75
AGUNet+YOLOv8	Frozen-backbone Training	0.712	0.643	0.79
AGUNet+YOLOv9	Frozen-backbone Training	**0.775**	0.695	0.74
FusionU10	Frozen-backbone Training+Fine tuning	0.768	**0.701**	**0.81**

Bold indicates the optimal result.

### 4.6 Detection performance on various occlusion conditions

In this experiment, to evaluate the performance of the proposed algorithm under different occlusion conditions, we divided the validation set of the M3FD dataset into four categories: no occlusion, non-pedestrian occlusion, pedestrian occlusion, and smoke occlusion. As shown in Table 8, the algorithm's performance varies significantly under different occlusion conditions, reflecting the impact of occlusion type on pedestrian detection performance. Firstly, in the no occlusion scenario, the algorithm showed the most stable performance, achieving a high precision (*P* = 0.934) and recall (*R* = 0.849). Additionally, the mAP@0.5 and mAP@0.5–0.95 reached 0.900 and 0.626, respectively, indicating that in obstacle-free environments, the algorithm can effectively detect pedestrians with high accuracy and robustness across multiple thresholds. For the non-pedestrian occlusion scenario, although the occlusion was not caused by pedestrians, both precision and recall slightly decreased to 0.921 and 0.807, respectively. Nevertheless, the mAP@0.5 remained at 0.904, demonstrating the model's strong robustness when faced with non-pedestrian occlusions. However, the slight drop in mAP@0.5–0.95 suggests that detection accuracy was affected to some extent under multiple threshold conditions. In the pedestrian occlusion scenario, the detection performance was more significantly impacted. Although precision remained at 0.911, recall dropped sharply to 0.665, indicating that the model's ability to capture targets weakened when pedestrians were occluded. Despite this, mAP@0.5 only slightly decreased to 0.896, and mAP@0.5–0.95 actually improved to 0.675, showing that the model can partially compensate for the negative effects of occlusion under different thresholds, exhibiting better robustness. In the smoke occlusion scenario, the detection results were the best, with recall reaching as high as 0.986, and mAP@0.5 and mAP@0.5–0.95 achieving 0.991 and 0.825, respectively. This indicates that despite the presence of smoke, the model can still accurately detect pedestrians, even exceeding expectations in such complex scenarios. Smoke had less impact on the model's detection ability, especially in terms of its performance on mAP@0.5–0.95.

**Table 8:** Pedestrian detection results under different occlusion scenarios.

**Scene**	**P**	**R**	**Pedestrian**	**mAP@0.5**	**mAP@0.5–0.95**
No occlusion	0.934	0.849	0.904	0.900	0.626
Non-pedestrian occlusion	0.921	0.807	0.902	0.904	0.614
Pedestrian occlusion	0.911	0.665	0.823	0.896	0.675
Smoke occlusion	**0.935**	**0.986**	**0.993**	**0.991**	**0.825**

Bold indicates the optimal result.

Overall, the type of occlusion has a significant impact on pedestrian detection. The model performed stably in no occlusion and non-pedestrian occlusion scenarios, while pedestrian occlusion greatly reduced recall. In contrast, smoke occlusion had minimal effect on detection performance, indicating that the proposed model has certain advantages in handling complex occlusion scenarios.

### 4.7 Limitations and future work

Despite the significant performance improvements demonstrated by the proposed FusionU10 model in pedestrian detection tasks, there are still some limitations that need to be addressed in future research. First, although AGUNet's lightweight design effectively improves inference speed and computational efficiency, the model's inference efficiency may still be insufficient to meet the needs of real-time systems when dealing with large-scale datasets. In complex scenarios, especially in densely populated areas or during long-term continuous monitoring tasks, balancing computational resource consumption and real-time responsiveness remains an issue that requires optimization. Secondly, FusionU10 relies primarily on multimodal fusion of infrared and visible light images to enhance detection performance. However, in extreme weather conditions (such as heavy fog or rain), both infrared and visible light information may be limited, which can reduce the model's detection accuracy. Future work could explore integrating other sensor data, such as LiDAR or millimeter-wave radar, with the existing multimodal fusion methods to enhance the model's robustness in extreme conditions.

Future research could proceed in the following directions: First, more efficient model compression techniques, such as knowledge distillation or model pruning, could be introduced to further reduce the model's computational complexity and optimize its real-time performance in large-scale scenarios. Second, new approaches to multimodal sensor fusion could be explored by integrating more diverse sensor data to enhance the model's adaptability in extreme environments. Additionally, combining transfer learning with self-supervised learning could reduce the reliance on large-scale labeled datasets and further improve the model's generalization capabilities and applicability.

## 5 Conclusion

The FusionU10 model introduced in this paper provides efficient and precise pedestrian detection by integrating multimodal data from infrared and visible light sources. Leveraging enhancements to AGUNet and YOLOv10 architectures, FusionU10 maximizes the advantages of multimodal information, achieving outstanding detection performance in challenging conditions, particularly in low-light and occluded environments where its robustness is apparent. Through the joint optimization of pseudo-color image generation and the object detection network, the model substantially improves both detection accuracy and processing efficiency. Experimental results indicate that FusionU10 delivers top-tier detection performance across multiple public datasets, validating its effectiveness in real-world applications. Additionally, the model demonstrates excellent adaptability to large-scale pedestrian detection tasks, offering reliable technical support for public safety management in densely populated environments. FusionU10 thus represents an innovative approach to pedestrian detection, establishing a solid foundation for intelligent tourism systems and real-time monitoring in complex settings.

## Data Availability

The original contributions presented in the study are included in the article/supplementary material, further inquiries can be directed to the corresponding author.
